# The Use of UV-Visible Reflectance Spectroscopy as an Objective Tool to Evaluate Pearl Quality 

**DOI:** 10.3390/md10071459

**Published:** 2012-07-10

**Authors:** Snezana Agatonovic-Kustrin, David W. Morton

**Affiliations:** School of Pharmacy and Applied Science, La Trobe Institute of Molecular Sciences, La Trobe University, Edwards Rd, Bendigo 3550, Australia; Email: d.morton@latrobe.edu.au

**Keywords:** artificial neural network, diffuse reflectance UV-Visible spectroscopy, pearl grading, pearl quality

## Abstract

Assessing the quality of pearls involves the use of various tools and methods, which are mainly visual and often quite subjective. Pearls are normally classified by origin and are then graded by luster, nacre thickness, surface quality, size, color and shape. The aim of this study was to investigate the capacity of Artificial Neural Networks (ANNs) to classify and estimate the quality of 27 different pearls from their UV-Visible spectra. Due to the opaque nature of pearls, spectroscopy measurements were performed using the Diffuse Reflectance UV-Visible spectroscopy technique. The spectra were acquired at two different locations on each pearl sample in order to assess surface homogeneity. The spectral data (inputs) were smoothed to reduce the noise, fed into ANNs and correlated to the pearl’s quality/grading criteria (outputs). The developed ANNs were successful in predicting pearl type, mollusk growing species, possible luster and color enhancing, donor condition/type, recipient/host color, donor color, pearl luster, pearl color, origin. The results of this study shows that the developed UV-Vis spectroscopy-ANN method could be used as a more objective method of assessing pearl quality (grading) and may become a valuable tool for the pearl grading industry.

## 1. Introduction

Pearls are unique organic gems, the only one created by a living organism and the only gems that do not require cutting or polishing before use. Pearl quality and hence, their value and beauty, is measured using various tools and methods that are mostly visual and often subjective [1]. Optical microscopes can be used to distinguish genuine, natural, and cultured pearls from fake pearls. In contrast to fake pearls that look smooth, genuine pearls show relief lines on their surface under 20× magnifications [2]. However, it is hard to differentiate between natural and cultured pearls. Natural pearls have a concentrically layered structure, whereas the inner structure of the cultured pearls varies according to the type of bead. Natural pearls can be distinguished from the cultured pearls using a hole drilled into the pearl. Through this hole the borderline between the nucleus and the nacre can be viewed in cultured pearls. However, for pearls without holes, methods using a lucidoscope or endoscope can be applied. These two apparatus need strong light gleaming through the pearls. Still, the most sophisticated and reliable way to identify the nature of pearls is examination using various X-ray techniques, *i.e.*, X-radiography (skiagram method), X-ray diffraction (lauegram method), and X-ray luminescence [3]. The last two methods can be applied to determine the thickness of nacre while the former is used for observing the diffraction pattern of the tested pearls. UV-Visible spectrophotometry has been used to distinguish between treated and untreated pearls and to determine their characteristic absorption/reflectance spectra that enables identification of species origin of cultured pearls [4]. Nevertheless there is no international standard method that can be used for overall pearl grading [2] and identical pearls may be graded differently by different suppliers. Pearls are usually initially sorted on the basis of species origin and then their quality is graded by size, shape, color, luster, nacre thickness, surface cleanliness and texture. 

The main aim of this research was to investigate the ability of Artificial Neural Networks (ANNs) to correlate diffuse reflectance (DR) UV-Visible spectra of selected pearls with their origin, species of the mollusk, donor color, possible color enhancing, and thus predict their quality. Once an accurate predictive ANN is established it can be used to evaluate pearl quality of an ungraded pearl based only on its UV-Visible spectrum.

Non-destructive methods of analysis, such as the diffuse reflectance method, allow rapid, sufficiently precise and reliable quality control with wide applications in many production systems. ANNs have been successfully used to classify spectra from various modalities including gamma ray spectroscopy [5], infrared spectroscopy [6], Raman spectroscopy [7], mass spectrometry [8], nuclear magnetic resonance (NMR) spectroscopy [9] and X-ray fluorescence [10]. Recent work by Karampelas *et al*. [11] utilized UV-Vis-NIR spectroscopy to identify absorption features associated with the various pearl colors. Also, a series of physical characterization methods (UV fluorescence microscopy, X-ray microdiffraction, backscattered electron imaging and X-ray absorption spectroscopy) were recently applied to Polynesian pearls to re-examine a theory of pearl formation [12].

The most important advantage of ANNs is in solving problems that are too complex for conventional methods. These problems include pattern recognition and forecasting. Common methods of data modeling are multiple linear regression (MLR) [13,14], principal components regression (PCR) and partial least-squares (PLS) [15]. In the multivariate case, such as in full spectrum analysis, when there is more than one independent variable, the general computational problem is to fit a line to a number of points. Full spectral classification techniques, PLS and PCR have been shown to be less successful in handling the low signal-to-noise ratio for complex mixtures. When applied to noisy data, they perform very poorly with the performance of PLS being usually better than PCR [16]. In such cases a more empirical approach such as the use of an ANN is more useful. Thus, the main reason selecting a more complex model, such as an ANN, over PCR or latent variables based models, such as partial least squares discriminant analysis (PLS-DA), is the presence of strong non-linearity in the data [17,18]. The ANN modeling technique has attracted increasing interest in recent years as a most promising candidate for classification and multivariate calibration problems.

## 2. Results and Discussion

Pearls, even though they are cultured, are organic gems. They are formed by a living creature, a living shelled mollusks that may produce a variety of pearl that is unique to the shell species. Pearl quality is measured by comparing size, shape, color, luster and surface complexion. Larger, unblemished, lustrous and spherical pearls are considered to be more valuable. Depending on the pearl oyster species, color is a more subjective indicator of value, with white South Sea pearls, especially those with a pink overtone, holding the greatest value of any marine pearl with similar quality characteristics [19]. However, only 30% of the cultured pearls can be categorized as high quality, due to the presence of blemishes or other defects. 

**Figure 1:**
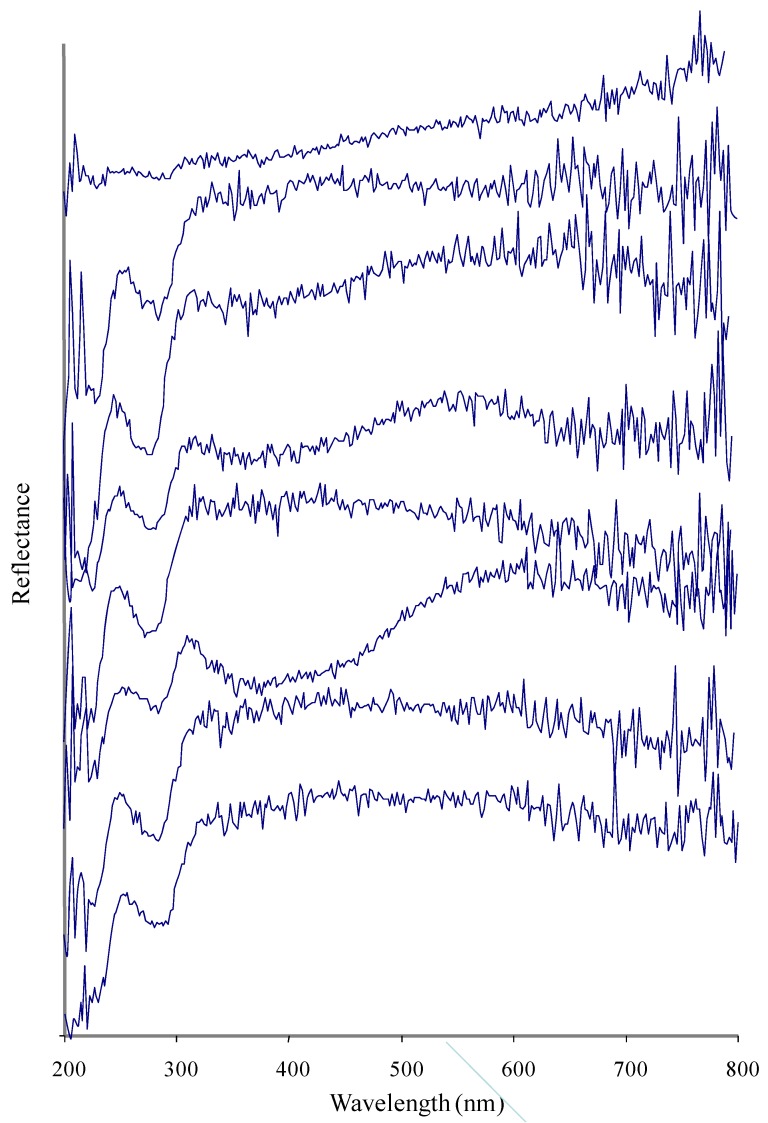
Spectral data (UV-Vis) of eight South sea pearls from *Pinctada maxima* (samples 1–8 in ascending order).

A UV-Visible spectrum of a pearl is a unique property and different pearls may show differences in their UV-Visible absorbance due to differences in nacre composition (Figure 1–Figure 4). The nacre, also known as mother-of-pearl (MOP), is a crystalline substance that creates the iridescent visual effect attributed to pearls. Nacre is intensely studied because its biologically controlled microstructure contributes to its remarkable strength [20,21,22]. Nacre is a biomineral composed mostly (about 95%) of thin layers of irregular polygonal platelets of aragonite, a crystal form of calcium carbonate (CaCO_3_) and even thinner layers (5%) of conchiolin (a scleroprotein), separated by elastic biopolymers (such as chitin and lustrin) [23]. Each layer of nacre is considered as being optically non-uniform [24]. Conchiolin is an organic protein, a dark-colored substance secreted by the mollusk during the initial phases of pearl formation that acts as a form of glue or adhesive. The conchiolin itself forms a matrix with irregular polygonal cells. This mixture makes the material very strong and flexible. Aragonite, a hard but brittle calcium carbonate crystal form, is considered to be a soft gem. However, when combined with the organic biopolymers and proteins that the mollusk naturally secretes the substance becomes remarkably strong, around 3000 times tougher than aragonite [25]. The microscale architecture of nacre resembles a three dimensional brick and mortar wall, where the bricks are densely packed layers of microscopic aragonite polygonal tablets (about 5–8 μm in diameter with a thickness of about 0.5 μm) held together by 20–30 nm thick layers of organic materials. The platelets in nacre from abalone shell and other gastropods are arranged in columns (columnar nacre), while the platelets in nacre from bivalves (mussels or oyster) are arranged in a more random fashion (sheet nacre). Remarkably, the arrangement and size of the platelets in nacre is highly uniform throughout the nacreous layer. Recent studies with synchrotron spectromicroscopy experiments [26] revealed that the aragonite platelets crystals in nacre are misoriented with respect to each other. This unique structural arrangement was a surprise and could play a role in nacre’s remarkable resistance to fracture. Quantitative measurements of crystal orientation, platelet size, and platelet stacking direction show that orientational ordering occurs not abruptly but gradually over a distance of 50 μm. Self-ordering of the mineral phase is fundamental in nacre formation. The iridescent appearance and quality of nacre has been attributed to light diffraction, both diffraction and interference (interaction between waves), or interference alone. The thickness of the aragonite platelets is about 500 nm, which is comparable to the wavelength of visible light (400–800 nm), leading to absorptive and reflective effects on different wavelengths of light and resulting in different colors of light being reflected when observed at different viewing angles. Thus, the iridescence color of the pearl varies with changes in both the angle of incident light and the angle of observation and is due to diffraction caused by the reflection grating structure of nacre.

**Figure 2:**
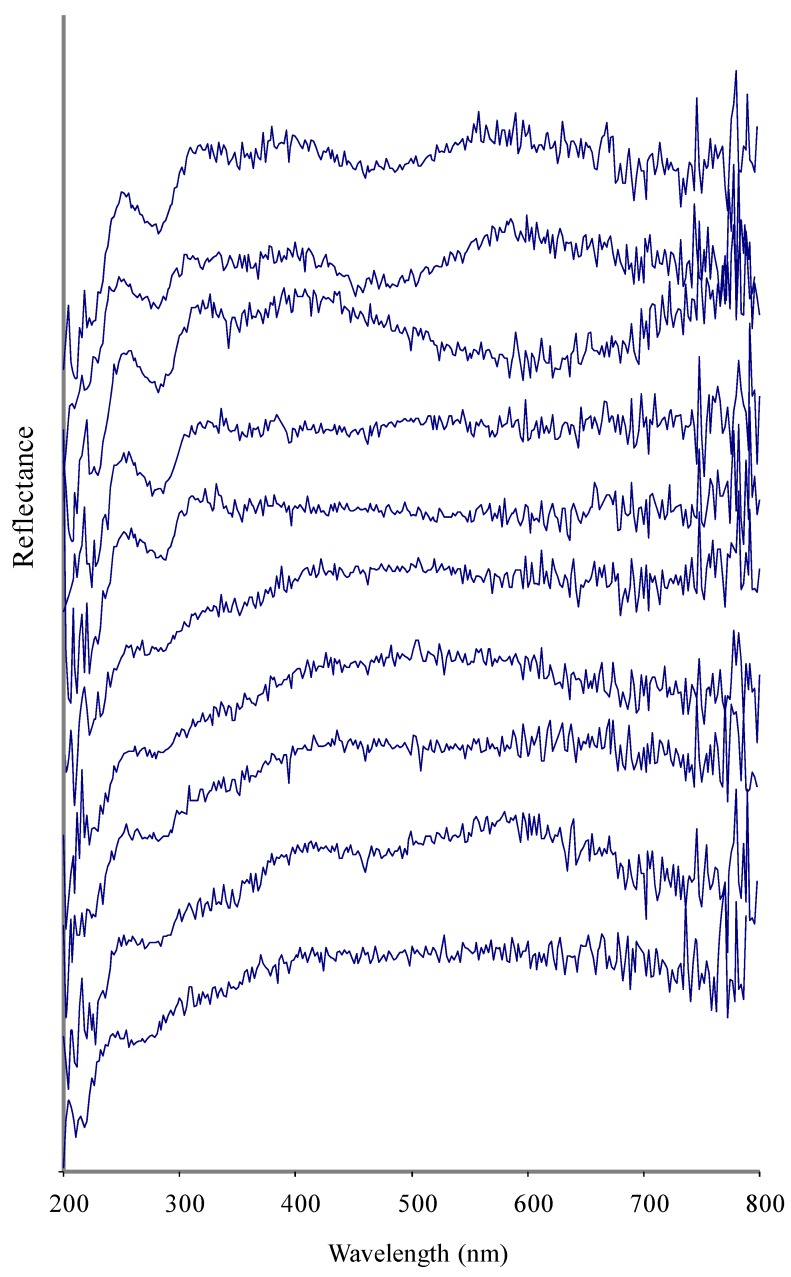
Ten superimposed UV-Vis spectra of Freshwater pearls from unknown species of Freshwater mussels (samples 9–19 in ascending order).

**Figure 3:**
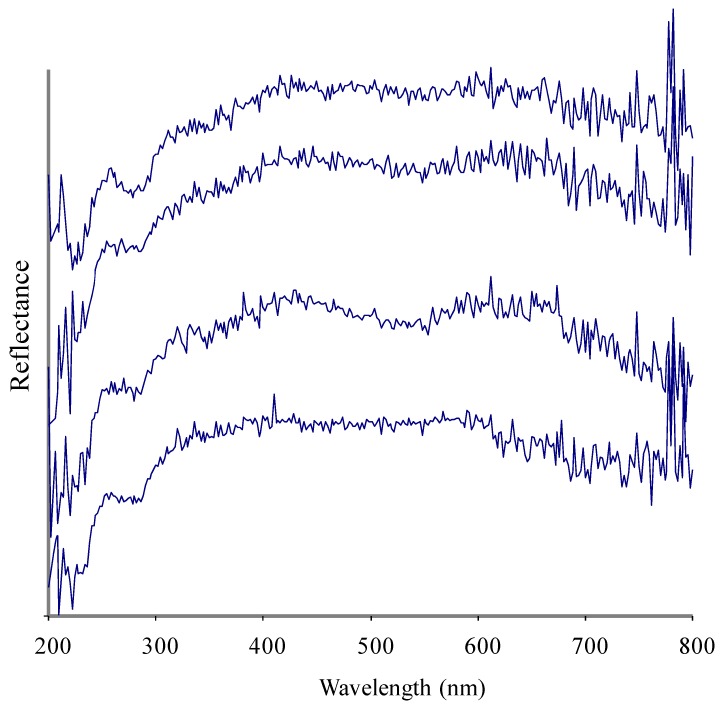
Four superimposed UV-Vis spectra of Akoya pearls from *Pinctada fucata martensi* (samples 20–23 in ascending order).

**Figure 4:**
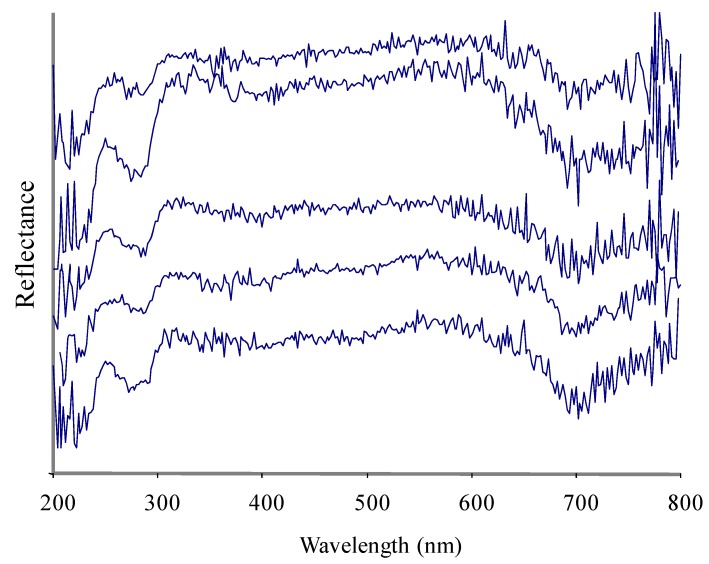
Five superimposed UV-Vis spectra of Tahitian pearls from *Pinctada margaritifera* (samples 24–28 in ascending order).

In this study ANN was used as the modeling tool to correlate unique UV-Visible spectral pearl patterns with pearl quality indicators. Artificial Neural Networks (ANNs) are superior to other modeling techniques in pattern recognition/classification due to the ultimately fine distribution and non-linearity of the regression. The ANN approach used here applied pattern recognition on the entire low-resolution UV-Visible spectrum and modeled all recorded peaks simultaneously. Twelve different ANN models were trained, tested and validated to classify pearls from their specific UV-Vis spectra (Table 1). Pearls were classified according to type, mollusk species, pearl locality, pearl quality (shape, color, luster, surface complexity), donor color and condition, recipient (host) color, possible color enhancing treatment and luster enhancing treatment.

**Table 1:** Developed Artificial Neural Network (ANN) models.

Pearl property	Model topology *	Pearl property	Model topology *
Pearl type	301-*8*-1	Surface complexity	301-*25*-6
Mollusc species MLP	301-*9*-4	Pearl shape	301-*8*-8
Pearl locality	301-*10*-4	Pearl color	301-*11*-12
Donor condition/type	301-*11*-3	Luster enhancing treatment	301-*8*-2
Recipient (host) color	301-*16*-4	Color enhancing treatment	301-*14*-3
Donor color	301-*10*-4	Pearl shape	301-*8*-8

* Number of inputs-hidden neurones-outputs.

The type of pearl and its species origin is perhaps the most basic factor to consider: whether the pearl is a freshwater pearl, an Akoya pearl, a South Sea pearl, or some other variety. Each type of pearl is created by a different species of oyster, usually living in a different region of the world and under different climatic conditions. All of these factors have an impact on the characteristic features of the resulting pearl. The developed ANN model for the pearls type had 301 inputs, 8 hidden neurons and one output. It successfully identified and classified different pearls with respect to their type (Table 2) and only one mistake was observed in the external validation set for the Akoya pearl sample coded 20 by predicting it to be Tahitian pearl. The possible cause of the error in prediction could be due to the smaller and insufficient number of pearl samples for Akoya (4) and Tahitian (5) pearls when compared to freshwater (11) and South Sea pearls (8), so that the trained networks may have been unable to completely distinguish between the different patterns for Akoya and Tahitian pearls. The most important commercial pearls are both natural and cultured, and include freshwater and saltwater (South Sea, Tahitian and Akoya) pearls. 

**Table 2:** Prediction of pearl type, mollusc species, and pearl locality using the optimised ANN model.

Pearl sample	Pearl type		Mollusc Species		Pearl Locality	
	Graded	Predicted	Graded	Predicted	Graded	Predicted
2	South sea	South sea	*P. maxima*	*P. maxima*	Bali	Bali
12	Freshwater	Freshwater	Freshwater mussel	Freshwater mussel	Zhuji, China	Zhuji, China
20	Akoya Pearl	Tahitian pearl	*P. fucata martensi*	Freshwater mussel	Japan	Japan
25	Tahitian	Tahitian	*P. margaritifera*	*P. margaritifera*	South Pacific	South Pacific

Cultured pearls are produced primarily by four main types of mollusk. Freshwater pearls are grown in various species of freshwater mussels, although most of the freshwater pearls are grown in *Hyriopsis cumingli*, while Akoya, Tahitian and South Sea pearls (saltwater pearls) are grown in oysters, the Akoya pearl oyster, *Pinctada fucata*, the black-lip pearl oyster *P. margaritifera* (Linnaeus), and the silver- or gold-lipped pearl oyster *P. maxima*. Akoya pearls are small saltwater cultured pearls from the Japanese pearl oyster *Pinctada fucata*. This type of oyster produces white or cream pearls and doesn’t naturally produce black pearls. Cultured pearls from *P. margaritifera* and *P. maxima* are generally referred to as South Sea Pearls, but for clarity, cultured pearls from *P. margaritifera* and *P. maxima* are referred to as Tahitian and South Sea pearls, respectively [27] and are distinguished by their color [2]. *Pinctada margaritifera* naturally secretes a black pigment, which, depending on the quantity, gives predominantly black pearls, with a basic color ranging from black to gray. South Sea pearls come in unique shades of colors ranging from a bright white to a deep yellowish orange. *P. maxima* is a large oyster also called the silver-lip/gold-lip oyster depending on the color of its shell lip. It produces pearls with a base color of gold or silver. 

In contrast, freshwater pearls cover a wider range of colors, size and shapes than any other pearl type. Their body color can be white, cream or colored (yellow, orange, pink and purple) and they may look remarkably similar to the Akoya pearls. Thus it did not come as a surprise that the model developed to classify mollusk species (*i.e.*, MLP 301-*9*-4) predicted freshwater pearl instead of *P. fucata martensi* (the Japanese pearl oyster that produces Akoya pearls) for the pearl sample, coded 20 (Table 2). 

Production of cultured pearls, commonly applied to pearl oysters, and now also to freshwater mussels, is a long process and requires a nucleus to be inserted into the mollusk in order for a pearl to develop. One possible way to improve pearl quality is by using mantle tissue cells or a graft from the donor oyster and to place it on top of the inserted nucleus. Saltwater pearls are made inside mature oysters, at least two years old. A small bead (nucleus) is inserted into the gonad of recipient (host) oyster along with a small piece of mantle tissue (saibo) from a sacrificed donor oyster [21], a process known as seeding or grafting [15]. The donor oysters are selected on the basis of their nacre color and luster since these characters may contribute to the quality of the resulting pearls [28]. The mantle tissue from the donor oyster then degenerates to a single outer epithelial cell layer which grows around the implanted nuclei to form a pearl sac, which is responsible for secreting nacre [29]. Subsequent proliferation of the donor mantle tissue forms the pearl-sac around the nucleus. This mantle tissue will secret the nacre or mother-of-pearl (MOP), cover the nucleus in concentric layers, and eventually form a cultured pearl over a period of about 2 years [21,30]. Until recently, it was not known if the donor oyster cells actually persisted in the pearl sac until time of pearl harvest. Genotyping the pearl sac, and comparing microsatellite alleles found with those from the corresponding host oysters, has shown that DNA originating from the donor oyster can still be detected in the pearl sac at the time of pearl harvest [31]. The cultivation process for freshwater pearls is similar except that freshwater pearls are cultivated in mussels and that during the implantation process, only a tiny piece of mantle tissue (nacre secreting) is inserted directly into the freshwater mussel. This acts as an irritant and begins the production of nacre which starts formation of the pearl. It is important to note that the cultivation environment of freshwater pearl mussel is greatly affected by artificial management, such as feeding and fertilization.

To optimize the quality of cultured pearl, pearl oysters must be treated appropriately to minimize stress during and after the pearl seeding procedure which may include forced opening of their shells and incision of the gonad prior to seeding. Relaxants have been used with pearl oysters to facilitate internal inspections, seeding operations for pearl production, and also to obtain saibo tissue from the donor oysters without killing the oysters. Anesthetics have also been investigated as a means of reducing stress and mortality of pearl oysters, to enable the removal of mantle tissue from donor pearl oysters during pearl seeding [32]. This potentially allows oyster donors that produce high quality pearls to be used as future breed stock. Furthermore, pearl oysters readily regenerate excised mantle tissue and so donor oysters that are anaesthetized for mantle tissue removal, rather than killed, could potentially be used for seeding on more than one occasion. Donor color and donor condition (relaxed or standard seeding) were successfully correlated with the UV-Visible spectral data with no mistakes, by the (301-*10*-4) and (301-*11*-3) ANN models respectively (Table 3).

**Table 3:** Donor condition and color, recipient color.

Pearl sample	Donor condition/type *		Recipient (host) color **		Donor color	
	Graded	Predicted	Graded	Predicted	Graded	Predicted
2	relaxed	relaxed	silver	yellow	silver	silver
12	standard seeding	standard seeding	White	White	White	White
20	standard seeding	standard seeding	White	White	White	White
25	standard seeding	standard seeding	Unknown (possibly black)	Unknown (possibly black)	Unknown (possibly black)	Unknown (possibly black)

* The mantle taken from donors was in relaxed condition with using anesthetic. ** Yellow consists of yellow to gold, and white as white or silver.

The quality of cultured pearl depends greatly on the selection of appropriate donor oysters. Thus, donor oysters are primarily selected by their nacre quality since it will influence the nacre quality of the resulting pearls [28]. The pearl color and complexation will also be strongly influenced by the donor oyster species. The donor tissue is chosen from oysters with attractive colors in the nacre lining their shells, which gives an indication as to the color of the resulting pearl. However, it is difficult to determine the donor color in a freshwater mussel because the color is different at different points in the nacre lining of its shell. It is common that freshwater pearls grown in a mussel exhibit different color, and color in a freshwater pearl also change in different layers (created at different growing phase). The factor(s) influencing color in freshwater pearls is not clear, and may be dependent on both the donor color and the composition of dissolved minerals present in the aqueous environment. The ANN model (301-*10*-4) was successful in predicting the donor color (Table 3). In the case of recipient (host) color, the best ANN model (301-*16*-4) only made a mistake for the South Sea pearl sample 2, predicting yellow instead of silver color. For pearl color, model (301-*11*-12) predicted various colors instead of silver and luster 2 instead of luster 1 for the Tahitian pearl sample coded 25 (Table 4). As mentioned before Tahitian pearls are cultured in the *Pinctada margaritifera* oyster, while South Sea pearl are produced in *P. maxima*. The pearl oyster species *Pinctada maxima* and its sister species *P. margaritifera* show unique pearl characteristics, particularly coloration, with *P. maxima* producing pearls with a base color of gold or silver, whilst *P. margaritifera* predominately produce black color based pearls. Cultured pearls from both *P. margaritifera* add *P. maxima* are generally referred to as South Sea Pearls. For clarity, cultured pearls from *P. margaritifera* and *P. maxima* are referred to as Tahitian and South Sea pearls, respectively [27].

**Table 4:** Pearl shape, color, luster and surface complexity.

Pearl sample	Surface complexity *		Luster **		Pearl color	
	Graded	Predicted	Graded	Predicted	Graded	Predicted
2	C1	B2	3	3	White	White
12	B1	B1	3	1	White	White
20	B1	B1	1	1	White	White
25	B2	B2	1	2	Silver with various overtone	Various color

* Surface complexity; B1: One to three very small blemishes in close proximity with the majority of the pearl surface being clear; B2: Three or more blemishes but with at least one clean face visible on the pearl; C1: Minor blemishes all over the pearl surface or one two large blemishes that affect over 70% of the pearl surface (wrinkled or scratched pearls fall into this category). ** Pearl luster grading factor; 1: mirror reflection luster; 2: somewhat mirror reflection; 3: chalky appearances.

When pearl grading, the appearance of the surface of a pearl is one of the most important characteristics in determining its overall desirability and value. Ideally, the pearl’s surface should be smooth, clean and shiny. It should have few, if any, bumps. The surface of a cultured pearl is examined in terms of the number, size, kind and location of the imperfection. In evaluating the imperfection, first, the number of imperfections is taken into account, that is, whether the pearl has clean surface, one spot or many spots. Surface complexity assesses the degree of blemishes or flaws covering the surface of a pearl. Blemishes may range from small spots to big chips or cracks or calcareous (chalky) bumps on the pearl surface [4]. Generally, the fewer surface blemishes a pearl have, the higher its quality. However, in grading South Sea pearls, big ridges forming rings (usually more than three rings) in a pearl is categorized as a circled pearl. Small spots (non calcareous) on the surface of a pearl are usually removed by polishing the pearl after harvesting. The error in the prediction of the surface complexity was minor. However, there was an issue with South Sea pearl sample coded 2, where the model predicted B2 (one to three very small blemishes) instead of C1 (minor blemishes all over the pearl or one or two large blemishes) (Table 4).

The pearl’s surface luster is critical in evaluating pearl quality. The unique luster of pearls depends upon the reflection and refraction of light from the translucent layers of the nacre. The iridescence that some pearls display is caused by the overlapping of successive layers, thus breaking up the light falling on, and reflecting from the surface. Pearls are usually white, sometimes with a creamy or pinkish tinge, but may be tinted with yellow, green, blue, brown, purple, or black. A pearl’s luster is a measure of its brilliance and the reflectivity of a pearl. It describes the reflectance of light from the pearl surface. High-quality pearls are bright and shiny, with high reflectivity (mirror-like reflectivity) while lower-quality pearls have a chalky or dull appearance. The luster of a pearl may be closely related to the homogeneity, light transmittance and thickness of the nacre. In general, saltwater pearls have a greater luster than freshwater pearls. Freshwater pearls and saltwater pearls differ in the type of luster. The difference is due to the type of mollusk used to produce the pearls and the thickness of the nacre. In appearance, Freshwater pearls are noted for a softer luster, a glow that comes from deep within the pearl as they are composed essentially of 100% MOP. The pearls in the freshwater mollusk start from virtually nothing (a small piece of tissue from a freshwater mussel). Freshwater pearls are cultured by tissue nucleation. Cultured pearls formed in a pearl sac in mollusks by inserting a piece (or pieces) of epithelial tissue. Since freshwater pearls are mantle-tissue nucleated they are entirely composed of nacre secreted by mollusks which gives a freshwater pearl outstanding luster. That also explains why it is rare to find a round freshwater pearl. Saltwater pearls have a more brilliant superficial luster. They are bead-nucleated and may only have 0.1 to 2 mm nacre over a bead nucleus. The model for the pearl luster grading factor was not as successful as the other factors predicted with two mistakes in the validation set by incorrectly predicting luster pearl samples 12 and 25 (Table 4).

Nacre thickness is the one of the most important elements of pearl quality. Important pearl characteristics, such as color and luster, are mainly determined by the nature of the pearl’s nacre. Nacre is the substance from which the pearl is actually created, and consists of layers of mother-of-pearl that grow layer by layer around the nucleus which is the core of the pearl. Pearls with nacre too thin or opaque tend to look chalky rather than iridescent. The thickness of the nacre layers is measured in millimeters (mm) and is strongly related to the length of the culture time after the nucleus implantation. The longer the pearl culture time, the thicker the nacre and the more valuable the pearl. If this layering is too thin, the core of the pearl will be visible through the gem’s surface. When a beam of light is incident into the nacre, it is strongly diffused by the layers. Thus, pearls normally appear a milky white without any iridescence color. Diffused light cannot cause interference; therefore, the layer structure of pearls and shells cannot produce the iridescence color. On the other hand, if the layer structure could produce interference, any pearl would show the iridescence color phenomenon. Although the diffused light contributes little to the iridescence color, it contributes to the body color of the pearl which represents the overall appearance color. Pearl colors range across almost the entire spectrum from white to black. A pearl’s overtone color(s) however is distinct from its basic color, and results in pearls in the same color category having very different looks and hues.

The colors of pearls are not merely due to the pigments that may be present in the pearl but also from the reflection and refraction of light [33], the nature of the material surrounding the nucleus [4], and the overall structure of the pearl. There are three main factors that contribute to overall pearl color; light phenomena (especially interference of incident light), pigments contained in the conchiolin, and the organic matter formed in the clearance between the inner surface of the nacre and outer surface of the bead. Color characteristics also differ according to the mother oyster species. The phenomenon of iridescence that shows glittering of various colors in a pearl is due to the interference and diffraction of light interacting with the specific structure on the pearl’s surface [2,28]. Iridescence is usually considered together with luster in pearl grading. Another color phenomenon is overtone or glow. An overtone color is a translucent color that may sometimes appear on a pearl together with its original (body) color [2]. However, it may alter the body color somewhat [4]. A pearl can be named silver-rose, which indicates that silver is the main (body) color and rose is the overtone. The developed model (301-*11*-12) made one mistake by predicted various colors instead of silver with various overtones for Tahitian pearl sample 25. The black-lipped oyster is the variety that produces the natural black color of Tahitian pearls. The most well known Tahitian pearls are those with the natural black color. However, they are found in a wide range of body colors which include: black, gray, blue, green and brown, and various hues. Body colors are enhanced by at least one overtone color. Overtone colors may include blue, gold, silver, pink and reddish purple. This is what causes the iridescent appearance of the Tahitian pearls. The color of South Sea pearls varies depending on which oyster the pearl comes from. The oyster from Northern Australia is the silver-lip oyster which tends to produce white South Sea pearls with a silver overtone. The gold-lip oyster which is commonly found in the sea around Indonesia, Thailand and the Philippines, produces yellow or creamy South Sea Pearls. Using ANNs pearl classification according to the color enhancing was 100% successful with no error in prediction (Table 5).

**Table 5:** Comparison of the classified pearls luster and color enhancing treatment and luster and color enhancing treatment predicted with ANN models.

Pearl sample	Luster enhancing treatment		Color enhancing treatment	
	Graded	Predicted	Graded	Predicted
2	no	no	no	no
12	likely	likely	possible	possible
20	likely	no	possible	possible
25	no	no	no	no

Pearl shape is mostly influenced by the shape of the nucleus, and in order to create a round pearl a round nucleus is required, although this does not always guarantee the formation of round pearls. It is not clear which factor could affect the shape of a freshwater pearl, and it is found that only a few freshwater pearls in fact have a round shape. Due to the complexity of possible pearl shapes a successful model that could predict pearl shape from its UV-Vis spectra is not likely to be able to be developed. 

## 3. Experimental Section

### 3.1. General

Twenty-eight graded pearl samples (Table 6) were obtained from commercial pearl farms. Eleven freshwater pearls were obtained from Zhuji (Zhejiang, China), 4 Akoya pearls were obtained from Japan, 5 Tahitian pearls were obtained from the South pacific and 8 pearls were obtained from a farm in Bali, Indonesia. These pearls were graded based on South Sea Pearl Grading System issued by Atlas Pacific, Ltd. [34] and had not been subjected to any color or luster enhancing treatments. 

Due to the opaque nature of samples, spectroscopy measurements were performed using diffuse reflectance (DR) UV-Visible spectroscopy. The diffuse reflectance UV-Visible spectra were collected using a Cary 50 UV-Vis spectrophotometer (Varian, Inc.) equipped with an external remote diffuse reflectance accessory (DRA) probe (Barrelino™, Harrick Scientific) which is able to scan an area less than 1.5 mm in diameter.

**Figure 5:**
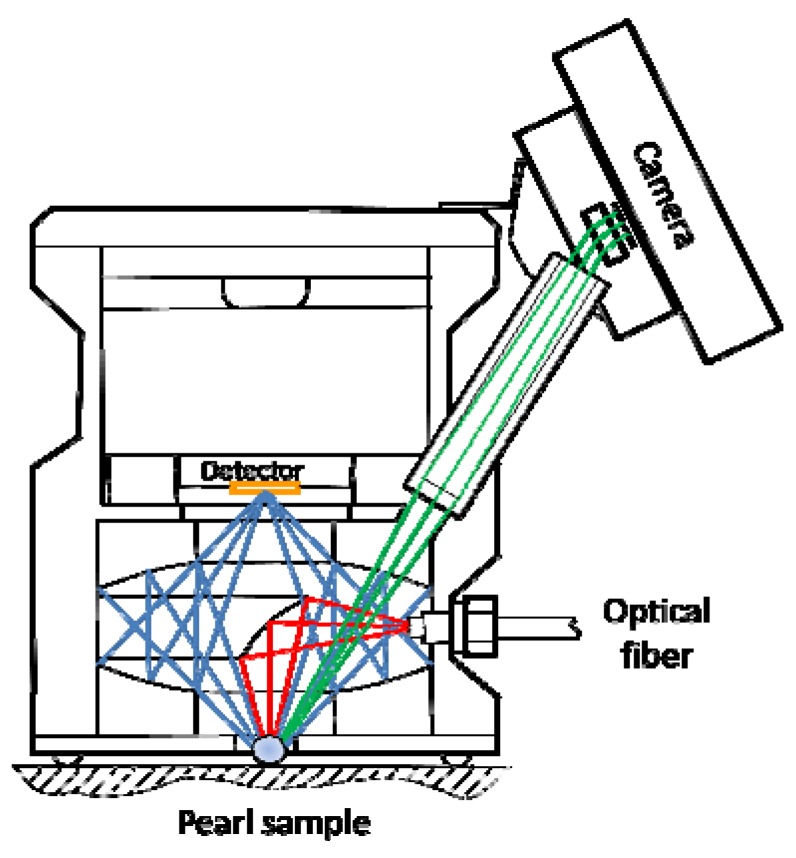
The Video Barrelino optical diagram.

The Video Barrelino (VB) probe (Figure 5) is a diffuse reflectance sampling probe designed specifically for the Cary 50 spectrometer. Monochromatic light from the spectrometer is carried by a 1.5 m long fiber optics cable and focused onto a sample into a spot less than 1 mm in diameter. A video camera is integrated into the accessory allowing direct viewing of the illuminated spot. There are two types of reflection, specular or mirror-like reflection and diffuse reflection [35,36]. Specularly reflected light is sometimes called shine or gloss. The VB probe is designed to collect only diffusely scattered light off the sample surface. The specularly reflected light is not collected for detection as it is not sample specific and tends to obscure the specific information contained in the diffuse component of the reflected light. The light reflected in all other directions is mostly the pure diffuse component. This radiation is collected and reimaged onto the built-in detector by two opposing coaxial parabolic mirrors. The focal point of the first mirror is on the sample; the focal point of the second mirror is on the detector. The bottom plate of the VB housing has an opening in the center to enable the light to reach the sample and the reflected light to be collected. The sampling plane is 1.5 mm below the bottom plate. Three Teflon balls imbedded into the bottom plate project out 1.5 mm and define the sample plane. For analysis, the accessory is simply placed onto the sample.

**Table 6:** Analyzed pearl samples.

Pearl sample		Pearl type	Purpose	Pearl sample		Pearl type	Purpose
1	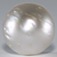	South sea	Training	15	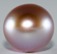	Freshwater	Training
2	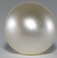	South sea	Validation	16	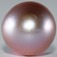	Freshwater	Training
3	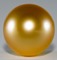	South sea	Training	17	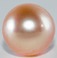	Freshwater	Training
4	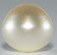	South sea	Training	18	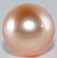	Freshwater	Training
5	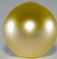	South sea	Training	19	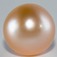	Freshwater	Training
6	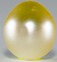	South sea	Training	20	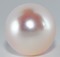	Akoya	Validation
7	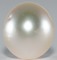	South sea	Training	21	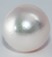	Akoya	Training
8	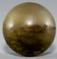	South sea	Training	22	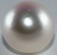	Akoya	Training
9	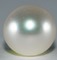	Freshwater	Training	23	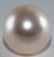	Akoya	Test
10	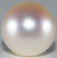	Freshwater	Training	24	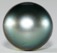	Tahitian	Training
11	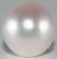	Freshwater	Training	25	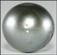	Tahitian	Validation
12	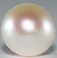	Freshwater	Validation	26	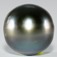	Tahitian	Training
13	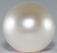	Freshwater	Test	27	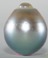	Tahitian	Test
14	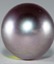	Freshwater	Training	28	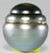	Tahitian	Test

UV-Visible spectra of pearls were acquired in the region 200–800 nm and 1500 spectral intensities were recorded during the data collection by averaging 10 scans for each spectrum and using appropriate baseline correction. Prior to scanning, the white level was calibrated with a wavelength reflectance standard (Labsphere^®^) in which approximately 100% reflectance across the entire spectrum is designated as a white reference standard. This standard sample was used to collect 100% reflectance, then the light source was blocked to obtain the 0% reflectance curve, commonly known as the “zero/baseline correction”. The spectra were acquired at two different locations on each sample to assess surface homogeneity, independent on shape of the pearl. In order to scan the pearls, each sample was put on a stand and scanned with the DRA probe connected to the UV-Vis spectrophotometer. The spectral data were then analyzed and the spectra were smoothed in order to reduce the noise in the spectrograms [35]. The 1500 absorbances were reduced into 301 averaged spectral values, each from consecutive 5 nm. The MS-Windows based artificial neural network software, Statistica. Data Mining, Artificial Neural Networks, Version 9.0 (StatSoft) was used for modeling the spectral data.

### 3.2. Artificial Neural Networks (ANNs)

ANNs are designed to simulate the way in which the human brain processes information and learns through experience with appropriate learning exemplars (in this instance spectral patterns and associated pearl quality factors). The behavior of a neural network is determined by the transfer functions of its neurones, by the learning rule, and by the architecture, itself. A categorical multilayer perceptron (MLP) Neural Networks was used in this study. In this type of model, information from inputs (UV-Vis spectral patterns) is fed forward through the ANN to optimize weights between neurons and to classify categorical outputs (pearl quality factors). A single hidden layer was used for simplicity, because there is little evidence to suggest that a larger number of hidden layers would improve performance. The output of the neuron is related to the summed input by a sigmoid shaped transfer function providing non-linearity to the model. During training, optimization of the network weights is made by back-propagation of error (e.g., the difference between the predicted and actual visually assessed pearl quality values), and the inter neuron connections (weights) are optimized so that the error in predictions is minimized. Once the network was trained and tested, it was given new input information (e.g., spectral data) to classify the categorical output (*i.e.*, to evaluate the quality of the new pearl of unknown origin).

From measured spectral data, training (70% or a 20 pearl data set), testing (15% or a 4 pearl data set) and validation sets (15% or a 4 pearl data set) were randomly generated. Averaged spectral intensities were used as inputs and pearl quality assessment parameters (pearls name, species, locality, luster, luster enhancing, color, possible color enhancing, donor condition, recipient, surface, and shape) were used as categorical outputs to train, test, and validate 12 different ANNs. The training set was used to actually train the network and the test set was used to monitor overtraining the network. The error in mapping the training values decreased as the number of hidden neurons was increased. During training and testing the number of hidden neurons and connections was varied from 0 to 30, in order to optimize network performance, with ANN performance tested after each addition. By increasing the number of hidden neurons, the ANN more closely followed the topology of the training group clusters on optimization. However, above an optimum level, adding more hidden neurons resulted in tracing the training pattern too closely and the system became over trained and lost its prediction capacity. Optimized models with 100% accuracy in classifying training and internal testing set were validated with the external set of compounds. Predictive performance for the validation sets was used to compare generalization ability of the models.

## 4. Conclusions

The results of this study show that the measurement of the UV-Visible spectra of pearls using Diffuse Reflectance (DRA) spectroscopy combined with an optimized ANN model, enabled accurate prediction of selected pearl quality parameters (their origin, species of the mollusk, color donor, possible color enhancing, donor color). This method for assessing pearl quality is quicker and faster than traditional methods and has the potential to result in fewer grading errors. The few prediction errors that did arise from the use of ANNs in our work are likely to be attributable to the relatively small number of pearl samples that were used to train the ANNs. The optimized ANNs developed in this work also enable the pearl quality to be more accurately assessed by those that do not have the experience and training to grade pearls using traditional and techniques methods which are often inherently subjective. Being less subjective, our method also has the added benefit of reducing the number of grading errors. From our preliminary results we believe this method offers the potential to become a valuable tool for the pearl grading industry.
